# Hypothalamic Obesity in Craniopharyngioma Patients: Disturbed Energy Homeostasis Related to Extent of Hypothalamic Damage and Its Implication for Obesity Intervention

**DOI:** 10.3390/jcm4091774

**Published:** 2015-09-09

**Authors:** Christian L. Roth

**Affiliations:** Seattle Children’s Hospital Research Institute, University of Washington, Department of Pediatrics, Center for Integrative Brain Research, 1900 Ninth Ave, P.O. Box 5371 M/S-C9S, Seattle, WA 98101, USA; E-Mail: christian.roth@seattlechildrens.org; Tel.: +206-987-5428; Fax: +206-987-7661

**Keywords:** hypothalamic obesity, craniopharyngioma, risk factors, neuroimaging, rodent models, inflammation, pharmacological interventions

## Abstract

Hypothalamic obesity (HO) occurs in patients with tumors and lesions in the medial hypothalamic region. Hypothalamic dysfunction can lead to hyperinsulinemia and leptin resistance. This review is focused on HO caused by craniopharyngiomas (CP), which are the most common childhood brain tumors of nonglial origin. Despite excellent overall survival rates, CP patients have substantially reduced quality of life because of significant long-term sequelae, notably severe obesity in about 50% of patients, leading to a high rate of cardiovascular mortality. Recent studies reported that both hyperphagia and decreased energy expenditure can contribute to severe obesity in HO patients. Recognized risk factors for severe obesity include large hypothalamic tumors or lesions affecting several medial and posterior hypothalamic nuclei that impact satiety signaling pathways. Structural damage in these nuclei often lead to hyperphagia, rapid weight gain, central insulin and leptin resistance, decreased sympathetic activity, low energy expenditure, and increased energy storage in adipose tissue. To date, most efforts to treat HO have shown disappointing long-term success rates. However, treatments based on the distinct pathophysiology of disturbed energy homeostasis related to CP may offer options for successful interventions in the future.

## 1. Introduction

Childhood obesity of any cause is a major risk factor for adult cardiovascular disease (CVD), the leading cause of death in the US [1]. In 2009–2010, 16.9% of US children and adolescents were obese (>95th BMI percentile) and rates of pre-diabetes and diabetes are still rising [2,3]. Approximately 84% of youth with severe obesity already have at least one CVD risk factor, and most adolescents with type 2 diabetes mellitus (T2DM) are obese [4]. In recent years, it has become clear that obesity can be associated with disturbed hypothalamic control of adiposity signaling peptides, such as leptin. The most striking examples of treatment resistant childhood obesity are observed in patients with dysfunctional hypothalamic signaling, such as in Prader-Willi syndrome, hypothalamic obesity (HO) due to craniopharyngioma (CP), or in subjects with deficient melanocortin signaling, leading to hyperphagia and excessive weight gain [5]. This review focuses on HO secondary to hypothalamic damage in patients with CP.

## 2. Clinical Manifestations and Risk Factors

### 2.1. Hypothalamic Obesity Syndrome

Excessive weight gain frequently occurs in patients with hypothalamic tumors and lesions, a disorder designated as hypothalamic obesity (HO). HO syndrome is characterized by fatigue, decreased physical activity, hyperphagia, decreased satiety, and severe obesity. All features are frequently seen in patients suffering from HO due to craniopharyngioma (CP) [6,7] as well as other causes of hypothalamic dysfunction and damage, such as other suprasellar tumors, inflammation, and genetic syndromes [6,8,9,10,11].

### 2.2. Hypothalamic Obesity in Patients with Craniopharyngioma

CP tumors are histologically benign (WHO Grade I), and are the most common suprasellar tumors of nonglial origin in children [12]. There are two peak age ranges: adolescence (10–19 year) and older adults (65–74 year), with 30%–50% of all CP cases occurring in childhood [13,14]. US data show 5.4% of all childhood (ages 0–19 year) brain and central nervous system tumors from 2006 to 2010 were CP [15]. Quality of survival is substantially reduced due to comorbid metabolic consequences [16,17,18,19]. Despite optimal endocrine management of hypopituitarism, severe obesity, a major risk factor for CP-related morbidity and mortality [20,21], occurs in about 50% of CP survivors [22,23,24,25]. Recent data show that hypothalamic involvement has major negative impact on long-term prognosis. Sellar masses with hypothalamic involvement were associated with lower overall survival and higher BMI at diagnosis and follow-up when compared with sellar masses without hypothalamic involvement [26]. The strongest weight gain occurs during the first 12 months following surgery [25]. While many patients report hyperphagia, recent studies have also reported an overall decrease in energy intake in patients with HO compared with controls [27,28]. Reduction in energy intake is offset by greater relative decrease in basal metabolic rate and physical activity related energy expenditure in HO patients, which might explain in part why weight loss attempts through caloric restriction and exercise has been largely unsuccessful [29].

CP patients are reported to be less physically active than controls with comparable body mass indices (BMIs) [28]. Hyperinsulinemia, leptin resistance and low leptin binding activity have been demonstrated in CP patients [29,30,31,32,33,34]. The degree of hyperleptinemia and hyperinsulinemia are inappropriate for the degree of obesity, and the metabolic rate and catecholamine levels are lower in CP patients, indicating a reduced sympathetic tone [6,27,28,30,35,36,37,38,39,40]. In recent studies, obese CP subjects had significantly reduced serum alpha-melanocyte-stimulating hormone (α-MSH) levels [35,41], higher baseline and post-meal insulin, and weaker post-meal gut hormone changes [27,32,37,39,40,42], indicating altered secretion of satiety-regulating hormones in CP. Reduced serum α-MSH levels indicate melanocortin deficiency, which might explain lower energy expenditure in peripheral tissues via reduced fat and muscle fatty acid oxidation [35,41,43]. All these findings underscore the concept of disturbed satiety signaling in CP patients and that leptin resistance and a deficiency of downstream mediators of leptin signaling are key features in HO pathogenesis.

### 2.3. Cardiometabolic Risk Factors

HO, caused by hypothalamic damage due to CP and its treatment, is one of the most refractory examples of childhood obesity leading to severe atherosclerotic CVD, T2DM and metabolic syndrome [44,45,46]. Patients with CP typically become obese and display more features of the metabolic syndrome compared to matched controls [34,44]. Overall, a 3–19-fold higher cardiovascular mortality has been reported [20,47], and a recent nationwide population-based study in Sweden demonstrated increased rates for cerebral infarction (seven-fold), death due to cerebrovascular diseases (five-fold), and type 2 diabetes mellitus (six-fold) in CP patients in comparison to the general population [21]. Thus, early and effective management of obesity is vital for these patients [48], which are more resistant to treatment than those with uncomplicated obesity [29,49,50,51,52,53,54,55,56].

### 2.4. Risk Factors for Excessive Weight Gain

Recognized risk factors for severe obesity include large hypothalamic tumors or lesions affecting several medial and posterior hypothalamic nuclei that impact satiety signaling pathways [25,51,57,58]. Structural damage in these nuclei often leads to hyperphagia, rapid weight gain, central insulin and leptin resistance, decreased sympathetic activity, low energy expenditure, and increased energy storage in adipose tissue [6,52]. Recently, Roth *et al.* developed a semi-quantitative assessment of hypothalamic damage on brain magnetic resonance imaging (MRI) to predict the risk for HO development in CP [25,59]. Beside neuroimaging criteria (see below), development of diabetes insipidus had been identified as an endocrine marker for increased HO risk [25]. However, no other hormonal abnormalities were identified that could serve as endocrine risk factors for HO development in patients who are adequately treated for endocrine disorders. In the same study, when comparing patients who developed HO *vs.* no HO, no differences were found in the rate of patients that received cranial irradiation in addition to brain surgery. Furthermore, in contrast to a previous study from Muller *et al.*, which demonstrated a correlation between perioperative dexamethasone doses and weight gain during the first year following craniopharyngioma surgery [24], there was no evidence that post-operative weight gain and perioperative dexamethasone dose were related. However, patients with HO more commonly had histories of complete tumor resections, which is in agreement with other studies showing that less invasive and hypothalamus-sparing surgical approaches decrease the risk for HO development [57,58,60,61].

### 2.5. Key Brain Areas Involved in Disrupted Energy Homeostasis

The hypothalamus is linked through direct synaptic connections to limbic systems that mediate motivation to eat and process reward [62,63]. Key regions thought to be the principal homeostatic brain areas responsible for regulating body weight are dispersed throughout the hypothalamus with important centers located in anterior (paraventricular nucleus, PVN), middle (arcuate nucleus, ARC, and ventromedial nucleus, VMN) and posterior (dorsomedial nucleus, DMN, and dorsal hypothalamic area, DHA) regions. The ARC contains two well-studied neuronal populations [64], one expressing proopiomelanocortin, a precursor of α-melanocyte stimulating hormone that inhibits food intake and stimulates energy expenditure; the other co-expressing agouti-related peptide and neuropeptide-Y, which both stimulate food intake and reduce energy expenditure. ARC neurons project to several brain areas of energy regulation, including the PVN, lateral hypothalamus, and dorsal hypothalamic nuclei [65]. The ARC expresses leptin receptors similar to the VMN and PVN [66]. The DMN contains gamma-aminobutyric acid and neuropeptide-Y expressing neurons as well as α-melanocyte stimulating hormone terminals projecting from the ARC. DMN projections contribute to the stimulation of thyrotropin-releasing hormone neurons in the PVN [67]. DMN, DHA, and VMN are key nuclei for locomotion and thermoregulation, mediating leptin-induced sympathetic activation of brown adipose tissue and energy expenditure [68].

Disruption of feeding circuits by damage to medial hypothalamic nuclei due to tumor, surgery or irradiation, has the potential to increase hunger by unopposed activation of orexigens from the lateral hypothalamus, or by blocking response to adiposity signals such as leptin as well as proopiomelanocortin in the arcuate nucleus from the medial hypothalamus. In either case, the result can involve unopposed activation of the lateral hypothalamic area, and thereby orexigenic peptides (melanin-concentrating hormone (MCH), hypocretin/orexin), or inhibition of anorexigenic peptides in the PVN (corticotropin-releasing factor, thyrotropin-releasing hormone, and oxytocin (OXT)), finally resulting in increased food intake and decreased energy expenditure. OXT is produced primarily in the PVN and supraoptic nucleus [69]. Leptin activates PVN OXT neurons through a melanocortin-4 receptor (MC4R) dependent mechanism, whereas leptin resistance is associated with downstream impairments in OXT release [70,71,72]. PVN OXT deficiency is seen in Prader-Willi syndrome and obese humans with mutations of the single-minded 1 gene [73,74]. CPs, by virtue of their hypothalamic location, have the potential to disrupt energy homeostasis at many levels, resulting in a complex clinical picture of HO syndrome characterized by severe obesity associated with leptin-resistance, fatigue, hyperphagia, impaired satiety, decreased sympathetic tone, and low energy expenditure [27,28,36,52,75,76,77,78].

### 2.6. Functional Neuroimaging in Patients with CP

Functional magnetic resonance imaging (fMRI) is a powerful tool for observing the human brain’s *in vivo* responses to stimuli. One recent study tested satiety responses in a small group of four adolescent CP patients *versus* four BMI matched adolescent controls [79]. Following a test meal, controls showed suppression of activation by images of high-calorie, energy dense food while CP patients showed trends towards higher activation in regions of interest including the insula, nucleus accumbens, and medial orbitofrontal cortex. These results indicate a dysregulated connection between the hypothalamus and corticolimbic circuits involved in food reward and that perception of food cues may be altered in patients with HO, especially after eating, *i.e.*, in the satiated state [79]. Another recent study tested 10 CP patients with known hypothalamic involvement and 15 age- and intelligence-matched control subjects (median age: 17.8 and 17.3 y) by fMRI using an emotional face recognition task [80]. CP patients exhibited an abnormal pattern of task-induced activation and deactivation in the anterior and posterior rostral medial prefrontal cortex indicating that hypothalamic damage impacts neural correlates in other brain areas that are important for memory retrieval such as the medial prefrontal cortex. Therefore, the fMRI approach is encouraging for conducting mechanistic studies of the brain in future investigating how distinct brain lesions affect (i) responses to food cues and satiety in brain areas that are involved in motivation to eat, food reward and control of appetite as well as (ii) behavior and memory functions.

### 2.7. Structural Neuroimaging in Patients with CP

In several studies using structural neuroimaging, large structural defects affecting several medial hypothalamic nuclei were associated with the highest risk of obesity and often cause treatment-resistant uncontrolled appetite and rapid weight gain [6,37,38,57,58,81,82,83,84]. Rapid post-operative weight gain was identified as a predictor for severe long-term obesity [24,25,38]. Although there is strong evidence that hypothalamus-sparing surgery, potentially in combination with radiotherapy, decreases the occurrence of severe obesity without increasing the local recurrence rate [57,58], development of severe obesity following CP is still a major problem requiring identification of risk factors for obesity development which would allow early interventions.

**Figure 1:**
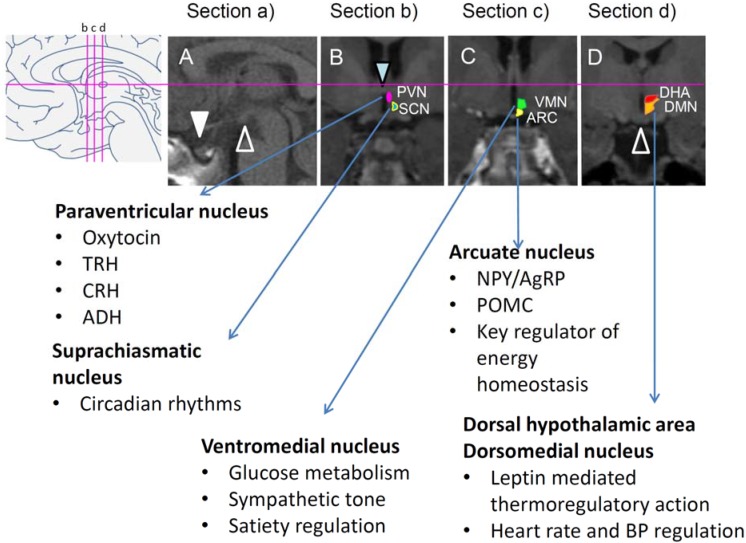
Standardized brain sections for assessment of brain lesions and hypothalamic nuclei that are critical for body weight regulation ((**a**) midline sagittal and (**b**–**d**) three coronal sections). Triangles point to landmarks of orientation: sella (**white**), anterior commissure (**blue**), and mammillary bodies (**white open**).

In the most recent study, a novel hypothalamic lesion scoring (HLS) system was used to assess hypothalamic damage and predict the risk for development of hypothalamic obesity, which can be used pre- and post-surgery [25]. In this study, damage was assessed in relation to distinct areas that contain key neurons of energy homeostasis in contrast to previous studies that included measurements of the two- or three-dimensional assessments of hypothalamic damage and compression [38,85,86]. Furthermore, it needs to be considered that the whole human hypothalamus has a volume of about 4 mL [51], which is thereby much smaller than many of the CP tumors before surgery (often greater than 20 mL). This scoring system qualitatively evaluates hypothalamic lesions using anatomical landmarks in sagittal and coronal MRI planes and does not require measurements or direct imaging of hypothalamic nuclei [25]. In addition, the HLS system differentiates between unilateral or bilateral damage of brain structures, as intact contralateral pathways could potentially compensate for deficient neurons and neuronal projections. Lesion scores are assessed in four standard images (Figure 1): (a) midsagittal (Figure 1A); (b) coronal section through the anterior commissure (Figure 1B); (c) coronal section midway between the anterior commissure and mammillary bodies (Figure 1C); and (d) coronal section through the mammillary bodies (Figure 1D). This procedure allows assessing seven criteria: (1) floor of the third ventricle which contains the arcuate nucleus; pituitary and pituitary stalk (Section a); (2) anterior hypothalamus including paraventricular nucleus which secretes antidiuretic hormone, corticotropin-releasing hormone, thyrotropin-releasing hormone, and oxytocin, as well as the suprachiasmatic nucleus, which is an important regulator for circadian rhythms (Section b); (3) medial hypothalamus containing the ventromedial nucleus, a key regulator for glucose metabolism, sympathetic tone, and satiety (Section c); (4) mammillary bodies (Section d); and (5) posterior hypothalamus containing the dorsomedial nucleus and posterior hypothalamic area which are important areas for thermoregulatory action, locomotion, heart rate and blood pressure regulation (Section d) [68]. In addition; (6) third and (7) lateral ventriculomegaly were assessed [25]. Compared to pre-surgical scoring, post-surgery lesion scoring correlated better with obesity outcomes [25]. Subjects with HO more frequently showed lesions affecting the anterior (paraventricular nucleus) and medial (includes arcuate and ventromedial nucleus) hypothalamus. However, the most robust weight gain was seen in subjects with lesions extending into the posterior hypothalamus containing the dorsomedial nucleus and dorsal hypothalamic area [25].

## 3. Studying Hypothalamic Obesity in Rats

### 3.1. Hypothalamic Lesion-Induced Obesity

As in humans, studies in rodents demonstrate that lesions affecting the PVN, VMN and the floor of the third ventricle, and thereby the ARC, bear the highest risk for impaired satiety and excessive weight gain [27,37,81,83,87,88]. However, conflicting results have been published as PVN lesions lead to marked stimulation of food intake but variable frequency of obesity [89,90]. VMH-lesioned rats showed lower activity levels and disturbed circadian rhythms, whereas rats with PVH lesions did not [89]. Rodents treated at neonatal age with monosodium l-glutamate (MSG), which destroys 80%–90% of the ARC neurons [91,92], develop neuroendocrine and metabolic abnormalities, resulting in a phenotype of adiposity characterized by GH deficiency [93], hyperinsulinemia [94], and hyperleptinemia due to leptin resistance [95]. Vagotomy decreased high insulin secretion induced in MSG-obese rats [96]. Although still widely used in lesion-induced obesity models [97,98,99,100,101,102], MSG-induced damage is not limited to distinct hypothalamic nuclei as it also penetrates the brain on other sites of circumventricular organs, e.g., subfornical organ and area prostrema, as well as eyes and pituitary [91,92,103,104,105,106]. In addition, MSG-induced obesity is not associated with hyperphagia [88]. Recently Roth *et al.* developed a novel rat model of combined medial hypothalamic lesions (CMHL) to study the pathogenesis of HO and test potential drugs for obesity treatment and prevention [6,41]. The characteristic phenotype of human HO could only be replicated when the ARC was included in the brain lesions [41,88]. The CMHL model has large lesions affecting several medial hypothalamic regions including the ARC, VMN, and also the DMN, leading to a more severe phenotype of HO and hyperphagia as well as melanocortin deficiency compared to smaller lesions and lesions of single nuclei [6,35,37,38,41,88]. As shown by different authors, the risk for gaining excess weight is particularly high during the immediate period following hypothalamic surgery [23,24,25]. During this critical time of rapid weight gain, brain inflammatory processes may be activated [107].

### 3.2. Inflammation as Potential Contributor for Disturbed Hypothalamic Signaling

Brain inflammatory responses are a hallmark of CP [107,108,109]. Increased interleukin (IL)-6 expression is observed in CP tissue and concentrations in cystic fluid reach levels 50,000-fold more than in cerebrospinal fluid. Increased IL-1α and tumor necrosis factor (TNF)-α are also observed in CP cyst fluid [110]. What remains unknown, however, is the role of inflammation in tumor- or surgery-related excess weight gain and food intake. To what extent do hypothalamic inflammatory or CP-elicited inflammatory processes impact energy homeostasis? There is emerging evidence that in rodents, high-fat diets cause metabolic inflammation leading to neuroinflammation, reactive astrocytosis and astrogliosis, increased cytokine expression, neural dysregulation of the hypothalamus, neurodegeneration, and defective adult neurogenesis [10,111,112,113]. In the hypothalamus, this leads to insulin and leptin resistance, specifically inhibitor of κB-kinase-β (IKKВ/NF-κB) activation and induction of suppressor of cytokine signaling (SOCS-3) [111,114,115,116]. Inflammation induced upregulation of SOCS-3, a marker of leptin and insulin resistance [117], can result in impaired ability of satiety signals, such as cholecystokinin-8, to activate neurons in the hindbrain and reduce food intake [118]. In rodent models, food intake can be inhibited by central suppression of IKKB [119,120,121]. These changes not only affect hypothalamic signaling, but also the regulation of energy homeostasis by downstream neurons [114,122,123,124], and may include reward pathways [125,126].

**Figure 2:**
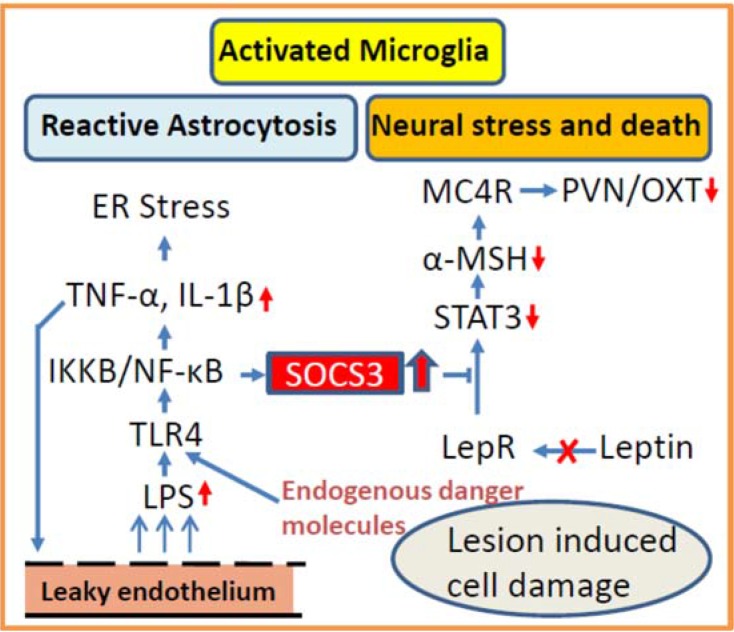
Schematic model of cellular and molecular components of neuroinflammation and repair mechanisms after brain surgery potentially affecting leptin receptor sites and melanocortin signaling resulting in disturbed energy balance. ER: endoplasmic reticulum; TNF-α: tumor necrosis factor alpha; IL-1β: interleukin 1 beta; IKKВ: inhibition of κB-kinase-β; NF-κB: nuclear factor-κB; TLR4: Toll-like 4 receptor; LPS: lipopolysaccharide; SOCS-3: suppressor of cytokine signaling 3; STAT3: signal transducer and activation of transcription 3; α-MSH: alpha-melanocyte-stimulating hormone; MC4R: melanocortin-4 receptor; LepR: leptin receptor.

Cellular components of neuroinflammation and repair after brain surgery include microglia and astrocytes with a maximum activation 5–7 days after insult [107]. Molecular components include pro-inflammatory interleukins, IL-1β, IL-6, IL-8, and TNF-α, which are produced by activated macrophages, T-cells, astrocytes, microglia and neurons [127], leading to neuronal dysfunction (see schematic overview in Figure 2). A deficient blood brain barrier is also observed in brain inflammation [128,129,130]. This can expose the brain to circulating endotoxins, such as lipopolyaccharides, which are ligands for toll-like receptor-4 (TLR4) [131]. TLR4s can also be stimulated by saturated fatty acids of a high-fat diet [113] and by binding endogenous danger molecules (heat shock proteins, hyaluronan, nucleic acids, and high mobility group box 1) in response to brain damage (Figure 2) [132]. TNF-α can induce endoplasmic reticulum (ER) stress [131]. The anti-inflammatory drug dexamethasone is frequently used perioperatively as a treatment for the prevention of cerebral edema and adrenal insufficiency, but bears the risk of excess weight gain. However, considering the mechanisms linked to inflammation listed above, abnormal weight gain can potentially be prevented by beneficial inhibition of inflammatory responses following hypothalamic surgery. The effects of other anti-inflammatory drugs could be tested in future studies.

## 4. Pharmacological Interventions

### 4.1. Rodent Studies

Drugs that mostly act through ARC signaling pathways (such as leptin) would not be likely candidates for reducing excess weight as the ARC is often involved in hypothalamic damage. More promising candidates are agents acting further “downstream” such as melanocortin (MC) receptor agonists that stimulate MC and are expressed in hypothalamic nuclei as well as extrahypothalamic and peripheral tissues [133], melanin-concentrating hormone-1 receptor antagonists that antagonize the strong orexigen melanin-concentrating hormone expressed in the lateral hypothalamus [134,135], and drugs that increase catecholaminergic signaling to stimulate energy expenditure [136,137]. Leptin resistance can be bypassed by MC 3/4 receptor agonist MTII in obese Zucker (fa/fa) rats, which are a genetic HO model with disturbed leptin signaling [138].

Recently, the CMHL rat model was used to test the efficacy of three pharmaceutical agents that act downstream of the mediobasal hypothalamus to reduce food intake and body weight. These agents include MTII, the glucagon-like peptide (GLP)-1 agonist exenatide and the psychomotor stimulant methylphenidate [139,140]. Both sham-lesioned and CMHL rats exhibited significant reductions in both food intake (lesion −20.8%, control −13.7%) and body weight when treated with exenatide relative to saline-controls [139]. Using a crossover design study, treatment with methylphenidate in both sham and CHML rats caused a significant decrease in food intake (CMHL −23%, *p* = 0.008; control −20%, *p* = 0.002) and body weight compared to saline-treated controls [140]. Finally, peripheral administration of MTII reduced food intake and body weight relative to sham-vehicle-treated controls (*p* < 0.05). Indirect calorimetry established that the effect of MTII was due to both a reduction in food intake, as well as an increase in energy expenditure [5,141].

### 4.2. Studies in Humans

Effective strategies to treat or prevent HO still need to be established as previous drug intervention studies were either too small or showed only moderate effects [29,49,50,51,52,53,54,55,56]. Treatment of hyperphagia and HO with the central nervous stimulant dextroamphetamine is reported to result in stabilization of weight gain but no weight loss [55,56]. Octreotide, a somatostatin analog, was reported to reduce both hyperinsulinemia and BMI in a double-blind placebo-controlled study; however, it supports weight stability only in patients with profound hyperinsulinemia and is limited by significant side effects [29]. Another attempt to treat hyperinsulinemia was combined diazoxide-metformin therapy; this was associated with reduced weight gain but significant side effects limited the enthusiasm after the initial pilot study [50]. A pilot study of caffeine and ephedrine given to three patients with HO resulted in a mean weight loss of 14% with two patients sustaining weight loss for at least two years; long term safety, particularly in the pediatric population, is unknown [49]. Reduction of energy expenditure in craniopharyngioma patients can be related to disturbed leptin signaling (see above) or hypothyroidism [51]. As patients with HO present commonly with panhypopituitarism including central hypothyroidism, it is necessary to treat central hypothyroidism appropriately yielding free thyroxine (FT4) levels in the upper normal range [142]. Tri-iodothyronine (T3) treatment aiming for supraphysiological T3 levels was considered by different groups [143,144]. Van Santen *et al.* [145] tested in one subject if switching treatment for central hypothyroidism to tri-iodothyronine (T3) monotherapy may be beneficial in increasing energy expenditure (thyroid hormone induced thermogenesis). However, T3 monotherapy was not effective as there was no change in BMI or energy expenditure assessed by sympathetic activity and brown adipose tissue thermogenesis.

Previous results of treating HO with a glucagon-like-peptide-1 receptor agonist (GLP1RA) in rats [139] and humans [146,147,148] are promising for conducting clinical trials in future. GLP-1 is secreted in response to ingestion of nutrients [149]. It is a key incretin that potentiates glucose-dependent insulin release and suppresses hepatic glucose output by suppressing glucagon secretion [150,151]. GLP-1 binds to receptors (GLP-1Rs) in the vagus as well as to appetite-related sites in the hindbrain (nucleus of the solitary tract, area postrema) and the hypothalamus (arcuate and dorsomedial nuclei) [152,153,154,155,156], which are areas that are not fully blocked from the peripheral circulation by the blood-brain barrier [150]. By binding to these receptors, GLP-1 functions as a satiety hormone, promoting reduced food intake and meal termination [153,154,155,156], as well as modulating activity in appetite- and reward-related brain areas [157]. Many anti-obesity drugs require intact hypothalamic signaling pathways for appetite inhibition, and, for such medications, weight reduction was poor for HO compared to uncomplicated obesity [54]. However, from a mechanistic standpoint GLPRAs might cause weight loss via intact hindbrain signaling pathways and thereby offer a desperately needed treatment option for HO, even in very obese HO subjects with severe hypothalamic damage [146,147,148]. Peripheral administration of GLP-1 or GLP1RA reduces blood glucose and energy intake in humans and rodents, and long-term treatment results in loss of body weight [158,159,160,161,162,163,164,165,166,167,168,169,170,171,172,173,174,175]. However, it is unknown whether GLP1RA treatment also affects energy expenditure and activity in subjects with hypothalamic lesions [176,177,178,179].

Further pharmacological approaches might include combinations of different drugs or newer anti-angiogenesis medication [180], in order to achieve persistent changes of energy homeostasis and negative energy balance.

## 5. Summary

In conclusion, hypothalamic obesity is a condition with extremely high morbidity. Mechanisms leading to the profoundly disturbed energy homeostasis are complex. Patients with hypothalamic lesions extending to nuclei located in the posterior hypothalamus have a particularly high risk for HO development. Standardized HO risk assessment using the HLS system may allow earlier identification of patients at risk and initiation of intervention strategies, which is critical as the strongest weight gain occurs during the first 12 months following surgery [25]. However, HO is extremely challenging to treat and effective strategies to treat or prevent HO still need to be established. Recent insights into the pathophysiology of lesion-induced HO might help to identify targets of promising obesity interventions in future.

## List of Abbreviations

α-MSH: alpha-melanocyte-stimulating hormone
ARC: arcuate nucleus
BMI: body mass index
CMHL: combined medial hypothalamic lesions
CP: craniopharyngioma
CVD: cardiovascular disease
DHA: dorsal hypothalamic area
DMN: dorsomedial nucleus
fMRI: functional magnetic resonance imaging
GLP-1: glucagon-like peptide-1
HLS: hypothalamic lesion scoring
HO: hypothalamic obesity
IL: interleukin
IKKВ: inhibitor of nuclear factor kappa-B kinase subunit beta
MC: melanocortin
MC4R: melanocortin-4 receptor
MSG: monosodium l-glutamate
MTII: melanotan 2
NF-κB: nuclear factor kappa B
OXT: oxytocin
PVN: paraventricular nucleus
SOCS-3: suppressor of cytokine signaling-3
TLR4: toll-like receptor-4
TNF-α: Tumor necrosis factor-α
T2DM: type 2 diabetes mellitus
T3: tri-iodothyronine
T4: thyroxine
VMN: ventromedial nucleus
